# Brill’s Disease

**Published:** 1914-02

**Authors:** 


BRILL'S DISEASE. Albert E. Roussel, of Philadelphia, *Md. Med. Jour.*, January, 1914, reports four cases and believes the fever identical with typhus which is probably not so rare as usually considered. This seems to be the consensus of opinion.

